# Reading intervention for students with intellectual disabilities without functional speech who require augmentative and alternative communication: a multiple single-case design with four randomized baselines

**DOI:** 10.1186/s13063-023-07452-4

**Published:** 2023-06-27

**Authors:** Line Britt Ulriksen, Marthe Bilet-Mossige, Hugo Cogo Moreira, Kenneth Larsen, Anders Nordahl-Hansen

**Affiliations:** 1 Faculty of Humanities, Sports and Educational Science, The University of South-Eastern of Norway, Notodden, Norway; 2 Institute of Education, ICT and Learning, Østfold University College, Halden, Norway

**Keywords:** Intellectual disabilities, Augmentative and alternative communication (AAC), Reading intervention, Phonological awareness, Letter-sound correspondence, Shared reading, Sight words, Decoding, Multiple single-case design

## Abstract

**Background:**

Literacy is one of the most important skills a students can achieve, as it provides access to information and communication. Unfortunately, literacy skills are not easily acquired, especially for students with intellectual disabilities who require augmentative and alternative communication (AAC). There are many barriers to literacy acquisition, some due to low expectations from parents and teachers and lack of evidence-based reading programs and reading materials adapted for AAC. Barriers as a result of extensive support needs is also a real factor. This trial aims to deliver reading instructions to 40 students with intellectual disabilities who require AAC and contribute in the debate on how to best support this population through reading instructions to maximizes their reading skills.

**Methodology:**

Forty non-verbal or minimally verbal students (age 6–14) with intellectual disabilities who require AAC will be part of a reading intervention with a multiple single-case design with four randomized baselines. The intervention period will last for 18 months and will commence in March 2023. The students will receive the intervention in a one-to-one format, working systematically with a reading material that contains phonological awareness and decoding tasks based on the Accessible Literacy Learning (ALL) developed by Janice Light and David McNaughton. All the teachers will be trained to deliver the reading intervention.

**Discussion:**

The reading material “Lesing for alle” (Reading for all) is based on and follow the strategies behind the research of ALL. The current trial will through a reading intervention contribute to move beyond only teaching sight words and combine several reading components such as sound blending, letter-sound correspondence, phoneme segmentation, shared reading, recognition of sight words, and decoding. The strategies and methods in use is built on the existing science of reading, especially what has been effective in teaching reading for students with intellectual disabilities who require AAC. There is limited generalizability of prior findings in reading-related phonological processing interventions to different populations of them who use AAC specially outside of the USA. More research is needed to understand how programs designed to improve reading skills across other settings understand the program’s long-term effects and to study the effectiveness when delivered by educators who are not speech language therapists or researchers.

**Trial registration:**

NCT05709405. Registered 23 January 2023.

**Supplementary Information:**

The online version contains supplementary material available at 10.1186/s13063-023-07452-4.

## Background

The importance of possessing literacy skills in today’s society should not be underestimated. Those who are literate benefit from increased access to education, employment [1], social interaction [2], mainstream technologies, and a wide range of information resources [3]. Literacy skills also have a powerful impact on communication and language development, on the improvement of cognitive development, and in the advancement of learning for individuals with intellectual disabilities (ID) and autism spectrum disorder (ASD) who require augmentative and alternative communication (AAC) support [4]. Literacy skills can enhance benefits even more for individuals with complex communication needs. Once an individual can read, the ability to communicate provides increasing possibilities for better communication with partners as well as larger access to a wider variety of opportunities [5, 6]. With improved communication, individuals will, to a lesser extent, need to rely on others to provide pictures or graphic symbols to express their ideas [4, 7]. Andzik and Chung [8] point out that individuals with complex communication needs may have intermitted or limited access to AAC during their school years, and their educational teams may not be equipped to provide AAC. Limited AAC may lead to a range of barriers throughout the life span [9].

Students with complex communication needs that require AAC are often excluded from phonological approaches to literacy. Browder et al. [10] found only 10% out of 128 studies focused on teaching reading skills that targeted phonics or phonemic awareness to students with intellectual disabilities considered to be moderate to severe in disability severity. Most literacy approaches require verbal production of speech sounds, which are particularly difficult for individuals with complex communication needs. Despite challenges with the spoken production of sounds, research has reported positive outcomes in participation in phonological interventions when they are adapted to meet the needs of students who require AAC [11-16].

A reading program that has been used in several studies is Accessible Literacy Learning (ALL). ALL is an evidence-based approach designed to teach reading skills to students with various intellectual disabilities, ASD, cerebral palsy (CP), and Down syndrome (DS) [3]. ALL combines a synthetic and analytical approach in reading instruction and is designed according to recommendations from the National Reading Panel [14].

## Previous relevant work

The body of research on reading instructions and intervention for students who require AAC is small [17], because it is conducted with a low prevalence population [11]. This contributes to little empirical evidence, and it can be difficult to measure the effectiveness of these programs, which are specifically designed to teach reading skills to students with intellectual disabilities, without functional speech [16].

It is also a challenge to build a cumulative knowledge base because of the wide variety of information and assessments reported in reviewed studies. The evidence-based research for reading interventions for students who require AAC consists of a small group of single-subject-design studies that use different measures and teaching strategies and demonstrate varying degrees of success [11]. Most studies have also focused on identifying sight words [10], but sight word instruction alone is not likely to result in a child with disabilities to learn reading [18].

A study by Fallon et al. [19] met the minimum standards of evidence established by the What Works Clearinghouse (WWC) [20] and provided strong evidence of intervention effects and moderate evidence of generalization. The result of this study, along with those of Millar et al. [21], were used to develop a literacy curriculum designed for children who use AAC [4]. The Accessible Literacy Learning curriculum [3] incorporated the instructional strategies and materials shown to be effective from these studies and is a good example of research informing practice [11].

A review of Yorke et al. [16] examined interventions from 1980 to 2019 that focused on improving basic literacy skills, such as phonological awareness (sound blending and segmentation), letter-sound correspondences, and decoding of simple words. They looked at the effect of studies with designs that were adapted to the needs of individuals with limited or absent speech and who used AAC. Seven of 22 interventions addressed ALL [4, 19, 22-25] and Westover, referred in Yorke et al. [16]. These studies showed an increase of 39% in total and large effects (Tau-U = 0.78) [16].

Another review, Bakken et al. [26], investigated the effects of reading and writing interventions for students aged 4–19 with intellectual disorder using randomized controlled trials (RCTs) and quasi-experimental designs (QEDs). The overall mean effect size from the reading interventions for trained reading was large (*g* = 0.95, 95% CI = 0.51, 1.28). However, the participants in these studies had varying levels of verbal skills, and only one study, Ahlgrim-Delzell et al. [27], specified that all the students were nonverbal and used AAC.

Barker et al. [11] identified eight articles in their review that reported teaching phonological awareness, individual word reading, and evidence-based literacy instruction for children with severe speech impairment who used AAC. Each study used a single-subject research design and was evaluated regarding whether it met Kratochwill et al. [20] minimum evidence standards (strong, moderate, or no evidence of an effect). Only five studies met criteria for strong evidence considering the effect of instruction on outcomes.

It was recommended that teachers chose an evidence-based reading program based on explicit teaching, corrective feedback, scaffolding, rewards, repetitions, and systematic teaching of phonological awareness and phonetic skills to ensure that students had opportunities to generalize their skills [7, 18, 28, 29]. Yorke et al. [16] also emphasized adapting an intervention so that verbal responses were not required, provided explicit practice with the skills, focusing on one to four foundational reading skills (directly corresponding to those measured), and included direct instruction procedures (i.e., introduction, model, guided practice, and independent practice with frequent positive or corrective feedback, in the intervention).

Reichow et al. [30] pointed out that additional experimental studies, in which there is efficacy of beginning reading interventions for children with intellectual disabilities, were needed. These additional studies could require several years of intensive intervention to demonstrate progress and allow for better understanding of possible outcomes.

It is important to describe participants characteristics prior to instructions, descriptions of the experimenter’s designed or modified standardized assessments, and the use of a strong research design. There is also a strong need for standardized assessments of both phonological awareness and reading that do not require speech responses [11].

Yorke et al. [16] point out that interventions should shift away from being administered by researchers towards administration by other communicative partners (e.g., teachers).

Future studies must consistently quantify participants’ cognitive, linguistic, and literacy levels and clearly describe the format and frequency of the intervention. It will be important to determine best practices related to intervention guidelines [16]. Future reading intervention studies for students with intellectual disabilities should include at least a 1-year follow-up in light of existing evidence of a fade-out effect within the first year or two following interventions [26].

## Methodology

### Aims

This trial investigates whether non-verbal or minimally verbal students with intellectual disabilities, who require AAC, can acquire foundational reading skills through instructions in phonological awareness (sound blending and phoneme segmentation), letter-sound correspondence, shared reading, sight words, and decoding by working systematically and explicitly with the reading program “Lesing for alle” (“Reading for All”). The adapted reading material has been prepared and designed in line on the basis of the research of the evidence-based reading program Accessible Literacy Learning (ALL) [3].

The research questions are as follow:

1. Is there a functional relationship between the use of “Lesing for alle” and increased accuracy of sound blending by students age 6–14 with intellectual disabilities who require AAC?
2. Is there a functional relationship between the use of “Lesing for alle” and improved acquisition of letter-sound correspondence by students age 6–14 with intellectual disabilities who require AAC?
3. Is there a functional relationship between the use of “Lesing for alle” and improved acquisition of phoneme segmentation by students age 6–14 with intellectual disabilities who require AAC?
4. Is there a functional relationship between the use of “Lesing for alle” and improved acquisition of recognition of sight words by students age 6–14 with intellectual disabilities who require AAC?
5. Is there a functional relationship between the use of “Lesing for alle” and improved acquisition of decoding by students age 6–14 with intellectual disabilities who require AAC?
6. Is there a positive and strong correlation between increasing skills from 1–3 to 4–5? Meaning, is there a transfer from lower-level skills (phonological skills) to decoding skills?

As this trial is a multiple single-case design, there were no comparator interventions chosen.

### Design

This study will be conducted with multiple single-case design with four multiple randomized baselines, where the shortest baseline has two assessments and the largest has five, and the intervention phase has 18 assessments across time. We have recruited 40 students with intellectual disabilities who require AAC.

**Fig. 1:**
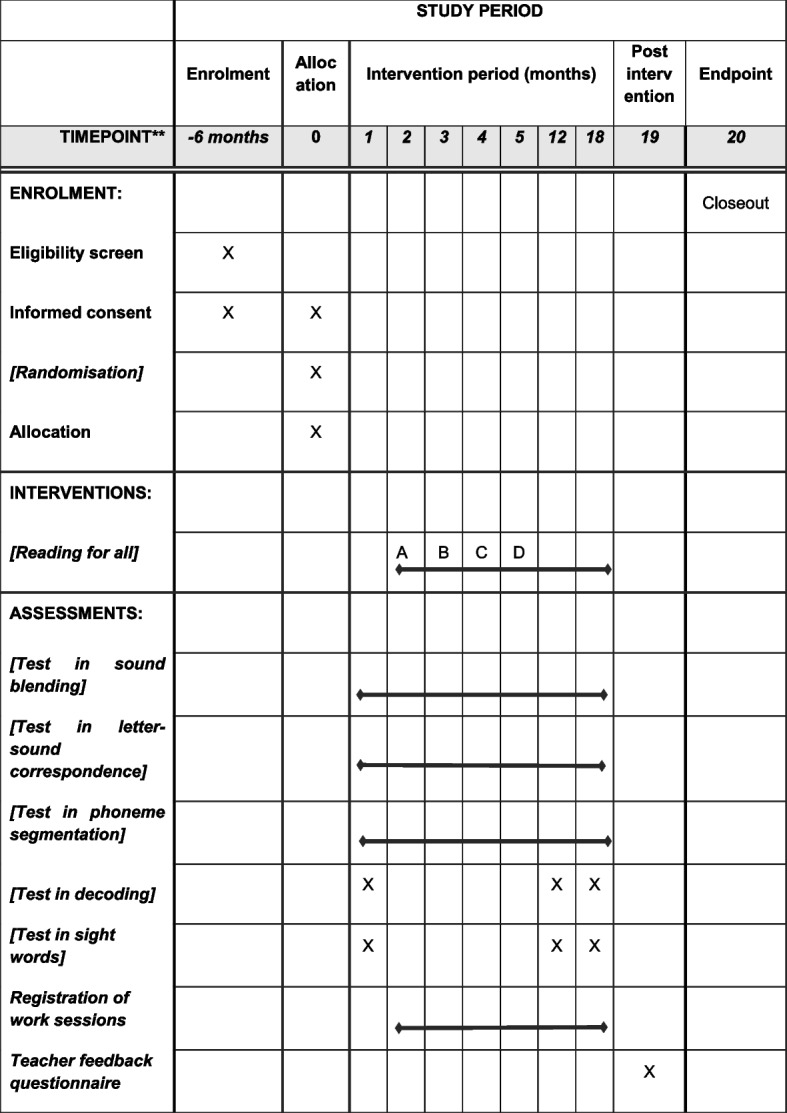
Assessments to be administered. This trial protocol is in line with standard protocol items: Recommendations for Interventional Trials (SPIRIT) 2013. Standard Protocol Items: Recommendations for Interventional Trials (SPIRIT) figure with timeline for recruitment, assessments, and interventions. Black lines indicate “conducted every month,” and X indicate “only conducted three times across 18 months, i.e., month 1, 12, and 18.” A, B, C, and D indicate the four randomized groups and when they start the reading intervention

To evaluate the effectiveness of the intervention effects in this study, the What Works Clearinghouse (WWC) single-case design technical documentation provides appropriate study analysis techniques [20]. Andzik and Chung [8] recommend that researchers follow these quality guidelines for single-case experimental studies to ensure internal validity of the study with reliable measures (Fig. 1).

### Sample size calculation and power

The power calculation is based on Hedges et al. [31] where the input parameters are as follows:

- Number the phases is equal to one (i.e., we have one baseline phase and one intervention phase)
- Intraclass coefficient correlation of 0.5
- Autoregressive effect 0.5
- Alpha error probability of 0.016 (giving we have three primary outcomes)
- Minimum number of observations per phase is three
- Minimum detectable effect size is a Cohen’s *d* of 0.5

Under those input parameters, our power is 75.84%; however, it is important to note that our study has an unbalanced design (different amounts of observations per phase resulting in the intervention phase being larger than the baseline). This is designed specifically to improve the adherence of the students and their families on the study. In our experience, being without intervention for more than 6 months (e.g., baseline phase) would reduce the interest and adherence of the participants in the trial. Therefore, our observable power will be superior than the above reported. The estimation of the power was conducted using the Power Analysis and Sample Size software (PASS) [32].

### Setting of the study

The intervention will take place in 12 schools from five different municipalities in Viken county in Norway. The participants are both in special schools, ordinary schools, and reinforced departments within ordinary schools. The teachers, familiar with the student, will deliver the instructions to the student during the school day. Each student has their own teacher/special education teacher who are responsible for their educational training. All teachers will receive individual training in test procedures, explicit teaching, and the reading material. The teachers will also be trained on how to score the tests and each work session. The main researcher will also be available for questions and guidance throughout the entire period.

### Intervention description

#### Recruitment

To recruit students, lectures were held at 12 schools, both ordinary schools, special schools, and schools with reinforced departments. The lectures were held in the period August to December 2022. The lectures emphasized informing about the reading program and the reading material “Reading for All” and about implementation and criteria for the reading intervention. Teachers, who had students within the criteria, registered themselves on an interest list, which the headteacher at the school passed on to the researcher. All the teachers were sent information letters and consent forms which they in turn sent to the parents, and all parents can contact the researchers by phone or email at any point in time during and after the trial. The teachers received their own consent forms. The principals at the respective schools collected all the consent forms and put them in sealed envelopes. They were then collected by the researcher and placed in a locked filing cabinet. The teachers who participate in the study will not be linked to the individual student, which help to strengthen the anonymity of the participants. The teachers and parents were asked to fill in the MacArthur-Bates Communicative Development Inventories (CDI) to document the individual student’s communicative competence. In February 2023, the teachers received training regarding the assessments and material. Baseline started in week 10 of March for all students.

#### Inclusion criteria

The inclusion criteria are as follows:

- Ages 6–14 years
- Diagnosed with intellectual disabilities, confirmed by multidisciplinary assessments carried out by the child rehabilitation service
- Does not have a functional speech, i.e., require augmentative and alternative communication (AAC) to understand and/or make oneself understood
- Must use AAC as their primary form of communication
- Cannot follow ordinary curricula LK20 [33] but have their own education plan

#### Exclusion criteria

The exclusion criteria are as follows:

- Students with severe visual impairment
- Students with severe hearing impairment
- Students who are able to decode single words and syllables
- Students with severe physical disability preventing the student from being able to point to answers with their fingers, unless they can respond with eye pointing or signs

#### Criteria for discontinuing or modifying allocated intervention

There will be no special criteria set for discontinuing or modifying allocated interventions. Given this is a non-pharmacological trial where the intervention is based on an educational approach with reading skills as outcome, we do not have special criteria for discontinuing or modifying allocated intervention. The parents and students are free to terminate participation at any time during the intervention.

#### Relevant concomitant care and interventions that are permitted or prohibited during the trial

Implementing the reading intervention will not require alteration to usual care pathways. The reading intervention is a complementary practice to what is offered in the schools. It is important to point out that in Norway, there is no evidence-based reading program for students who require AAC.

### Intervention

#### The reading material

The reading material “Lesing for alle” is based on the research of ALL [3]. The purpose is to work systematically with the various components within phonological awareness skills (sound blending and phoneme segmentation), letter-sound correspondence, shared reading, recognition of sight words, and single-word decoding. All the instructional activities have been adapted, and oral/spoken responses are not required for the students. The students can use alternative methods, such as signs, pointing at symbols, or pointing with their eyes. Evidence-based teaching based on the following: direct and systematic instruction [14, 16], explicit instruction [4, 14, 16], scaffolding, immediate and corrective feedback, cumulative review, and practice are used [3].

The reading program operates with six essential components:

- *Sound blending*, i.e., ability to build words from individual sounds by blending or combining sounds in sequence ([3], p. 71). Light et al. [4] argued that sound blending skills are important in learning to decode new words.
- *Letter-sound correspondence*, knowledge of the sounds represented by each of the letters and the letters used to represent different speech sounds [3]. Early letter knowledge is one of the factors that most strongly predicts reading skills later [34]. Being able to associate letters with sounds quickly and sounds with letters will help students to read and write [14].
- *Phoneme segmentation* is about dividing a word into sounds, counting sounds, pronouncing each sound clearly and separately, and the ability to listen out and break down words into single sounds [35]. Carnine et al. [35], Hulme et al. [36], and NRP [14] referred to research that described that phonological awareness correlated strongly with the acquisition of skills in reading and spelling. Browder et al. [37] emphasized that students with intellectual disabilities benefited from teaching phonemic awareness, but it was important that the phonemes were visually presented in the form of letters and pictures, so they could respond without functional spoken language.
- *Recognition of sight words*, ability to read/recognize a word without sounding it out [3]. Both phonological reading and word recognition are necessary for acquiring reading skills, and reading interventions are most effective when combining sight words with teaching decoding [10].
- *Single-word decoding*, the ability to recognize letters in words, associate them with correct sounds, and pull them together to the word the letter sequence represents [38, 39]. Decoding depends on letter knowledge and phonological skills [40, 41]. Good letter knowledge is a prerequisite for understanding and mastering the alphabetic principle, based on sounds represented by graphemes consisting of one or more letters [4].
- *Shared reading* is about the ability to use acquired skills and skills under development. Once students have developed basic skills in decoding simple words, it is important to use these skills in the context of meaningful and fun reading experiences [3, 4]. Browder et al. [42] claimed that shared reading helped to promote early reading skills for students with intellectual disabilities and is of importance as ordinary reading aloud is not as effective as explicit instruction in shared reading for this population of students [43, 44].

Emphasis will also be placed on working with additional activities such as flash cards and working on conceptual learning of the words they encounter in the reading program. All teachers get an overview of all the words that belong to the various levels in the reading material. It will be important to ensure basic comprehension skills on word level.

#### Design of the material

The tasks in sound blending, letter-sound correspondence, phoneme segmentation, sight words, and decoding are divided into four rectangular squares with a black frame in an A4 format. There is a symbol, letter, or word in each square. There is a corresponding instruction on each back of the assignment. The instructions lay down guidelines for what the teacher should say and how. The correct answer, target sound, or target word is marked in green so that the teachers can see what is correct. The instructions are short to adapt to the amount of verbal instruction the students can handle. Words and letters are written in small letters, with the font Arial, and the font size varies according to the different tasks. The tasks in “Shared reading” are made with images taken from the ALL App [45]. Through levels 1, 2, and 3, students encounter many words and symbols, about 350. The words are taken from McArthur-Bates Communicative development Inventory [46]. The entire material is made in the program InPrint and consists of Wigdit symbols [47]. Wigdit symbols are an English symbol layout that was created to support the reading and writing development of children who had difficulty cracking the reading code [48].

The material contains the following:

- *Level 0*. One folder (with about 60 tasks in explicit instruction in sound blending, letter-sound correspondence, shared reading, phoneme segmentation, sight words, and single-word decoding). Explicit instruction involves the teacher specifically identifying the skills for students to be taught in the lesson and then directly modeling the skill [49]. The current intervention will make use of explicit instruction through progressive scaffolding, which allows for systematically modeling of the concept, guided practice, and eventually to independence.

During the first step, the teacher is modeling for the student what will be required of them in each task and the student is a passive observer. The only expectation of them is that they are paying attention to the teacher. The teachers must follow the various scripts in the various reading components. The first step in the task is for the teacher to name all the four symbols and say “I’m going to say a word, listen to the word.” The teacher says the target word (for example seal). “Ssseeeaaalll,” the teacher says the word gradually faster, “sseeaall,” and finally say it normally, “seal.” Then, the teacher says “now I look at the symbols” and choose the symbol for “ssseeeaaalll.” The teacher points to the symbol for seal and at the same time says “ssseeeaaall” is a seal.”

During the second step, guided practice, the student is expected to be active in the lesson and is encouraged to point to the target item as well. In this step, the students get support to complete the tasks correctly together with the teacher. The teacher starts by saying “let’s do the task together.” It is the same script as in the modeling phase, but the teacher encourages the student to do it together.

During the final step, independent practice, students must perform independently. Teacher emphasizes and says “now it’s your turn, tap the glue icon.” If the student points to the correct symbol, the teacher says “good, g-llluuueee is glue.” The teacher points and confirms that the student has chosen the correct symbol. If the student answers incorrectly, the teacher corrects immediately and says “You pointed to xx (wrong answer), we have to find glue.”

- *Level 1*. Eight folders (with about 120 tasks in sound blending, letter-sound correspondence, and shared reading in each folder)
- *Level 2*. Thirteen folders (with about 120 tasks in sound blending, letter-sound correspondence, phoneme segmentation, single-word decoding, and shared reading in each folder)
- *Level 3*. Ten folders (with about 150 tasks in sound blending, letter-sound correspondence, phoneme segmentation, sight words, single-word decoding, advanced word decoding (for example, words with multiple consonant accumulations), not phonetic word pictures, recognition of high-frequency words, and shared reading in each folder)

There are clear instructions on the back of each task, so the training is carried out in the same way regardless of who is teaching the task. This also helps to create structure and predictability for the students. At the same time, the material is adapted so each student can use their alternative forms of communication. It is a goal that the students will receive instructions daily for about 30 min. The intervention will take place for a minimum of 1 year. The teachers must register each work session in registration forms. The forms are designed so that the teachers can select options 1, 2, 3, or 4, depending on how the student responds. The teachers must also register the time used with each work session and whether the student worked with explicit instructions or the reading tasks.

#### Assessment of the outcomes

A registration form has been developed to use during the assessment and data collection of outcomes. The form is designed so the teacher can click whether the student answers options 1, 2, 3, or 4. It is the student’s teacher who will carry out all the tests. The teacher must register correct or incorrect responses during the assessment. Only the teacher and the student will be present during the testing, and all test situations will be filmed. Each student will receive an individual follow-up plan that will describe in detail when tests will be carried out and when the intervention will start. Within each phase, 20% of the recorded films will be randomly selected and evaluated by two external raters at item-level across the five primary outcomes. Agreement between the two raters and the teachers will be checked, and in case of agreements below 80%, all the films will be checked.

#### Primary outcomes

Primary outcomes will be assessed 18 times in equidistant time points, every month, across 18 months. All the below described tests were developed by the first and second author. To the best of our knowledge, there are no such available tools in Norwegian to assess this population who uses AAC with demonstrated evidence of validity. They are centered on the following phonological awareness skills domains:

1. Sound blending [score ranging from 0 to 10] measured by adapted tests in line with the reading material “Lesing for alle.” Researcher-designed test. Testing in sound blending involves testing the student’s ability to combine individual sounds into words. The students will see a sheet of paper with four symbols. In the instructions, the target word will be presented by expanding the individual sound in the word. For example, “sss-uuu-nnn.” The students have to listen to the sounds, put the sounds together in their head, and then point to the symbol that belongs to the word. This test consists of 10 tasks. The teacher will make no comments on the accuracy of student’s selections but just comment “good work” for the motivation.

Example of one test task in sound blending:



Wigdit symbols [47].

2) Letter-sound correspondence [score ranging from 0 to 25] measured by adapted tests in line with the reading material “Lesing for alle.” Researcher-designed test. Testing in letter-sound correspondence involves testing the student’s ability on the correspondence between the sounds of speech (phonemes) and the written letters that represent speech (graphemes). The instructor asks the student to choose, for example, the letter sound “*a*.” The student must then discriminate between the target sound and three others. This test consists of 25 tasks.
3) Phoneme segmentation [score ranging from 0 to 10] measured by test in line with the reading material “Lesing for alle.” Researcher-designed test. Testing in phoneme segmentation tests the student’s ability to segment the first phoneme of the word (e.g., *s* for *sun*). Then, the student must select the symbol for the word that begins with the target phoneme. The student must discriminate between four symbols. The instruction asks the student to find the word that starts with, for example “*a*.” This test consists of 10 tasks.

#### Secondary outcomes

The time frame is as follows: two time points during baseline phase, two during intervention (months 1, 12, and 18). In the same way as described above, we have a lack of evidence in terms of validity in the following tests in Norwegian, and those assessments were specially developed for this population. Part of this study is focused on providing evidence based on other variables [50].

1. Recognition of sight words [score ranging from 0 to 10] measured by adapted tests in line with the reading material “Lesing for alle.” Testing in sight words measures the student’s ability to recognize words learned by sight, i.e., words that are difficult to decode. The student must discriminate between one target sight word and three others. The three other words consists both of other novel sight words, words that start with the same first sound as the target word and words of similar form. This test consists of 10 tasks with consonant–vowel-consonant (CVC), CV, VCVC, CCVC, VCCVC, and VCC words.
2. Decoding [score ranging from 0 to 20] measured by adapted tests in line with the reading material “Lesing for alle.” Testing in decoding tests the student’s ability to (1) recognize the letters in the word, (2) associate each letter with its corresponding sound, (3) hold these sounds in sequence in working memory, (4) blend the sounds to determine the word, (5) retrieve the meaning of the word [51], and, finally, (5) select the symbol that represents the word. The students must discriminate between four symbols, where one symbol represents the target word, and the other three consist of words with the same first sound, novel words, and words with similar forms. This test consists of 20 tasks with both CVC, VCC, and CCV words, for example, *bil* (car), *ost* (cheese), and *tre* (tree). Ten words will be familiar from the reading material, and 10 words will be novel; the words are thus not being worked explicitly through the reading intervention.

#### Data collection by survey

Questionnaires will be made in www.Nettskjema.no, which is secure for storing confidential data. The research team will have access to the material, and answers will be deleted at the end of the project.

#### Plans to promote participant retention

The teachers in the study are followed up closely throughout the process, and they receive emails three to four times each month with reminders and motivating comments and always before and after testing. The first author has close contact with the teachers and delivers memory cards for the video cameras to all schools before testing and collects the memory cards immediately after the testing is finished. The first author is also available via email and phone throughout the intervention period and visits the schools when necessary and at request of the teachers. The teachers also send monthly summaries by email (anonymously) so that the first author has an overview of what and how much is being done at any given time. This helps us to intervene early if someone is lagging behind in the study or do not adhere to the protocol. If participants drop out of the study for legitimate reasons such as illness, moving, behavioral changes, or that parents want to withdraw them from the study, there is no plan for further follow-up. Data will be registered as missing data.

Regarding the participants retention (students), we have the following strategies: it is a goal that the students do the work sessions once per day. The teachers have been instructed in how to motivate the students, by using reinforce that students prefer. It is also important to involve the parents as a strategy for adherence, e.g., sending updates to the parents so they also can motivate their children to participate in the intervention.

#### Procedure

Before the start of the intervention, the researcher will ensure that all the teachers have the necessary materials, video camera with memory card, and tripod. The researcher must also ensure that all the teachers can use the video camera.

#### Data management

Data will be anonymized and stored on a restricted shared drive at the University of South-Eastern of Norway (USN) at USN Safe, to which only the research team will have access. Per the informed consent, anonymized data will be retained permanently (except in the case of withdrawal during the study) and may be released to other researchers. Identifying data (e.g., video films) will be deleted after 4 years. Data entry will be completed and checked by the research staff. Video data will be sent encrypted to the USN’s secure storage devices, accessible only by the research team. Physical data will be stored in locked cabinets within the Østfold University College and accessible only by the research team. Physical anonymized data, which describes the weekly summaries from the teachers, will be stored on a password-protected laptop computer.

#### What data will be generated

There will be data that will say something about the student’s age, gender, diagnosis, IQ, AAC aid, vocabulary and communicative competence (through results from CDI, McArthur-Bates Communicative Development Inventories), results from the assessments, and time used during the intervention. The data will also provide information about the teacher’s and student’s experiences of being part of the project (social validity).

#### How the data should be described

Each student will receive a specific number (subject ID) throughout the intervention. The list of names and numbers will be stored in a locked filing cabinet in a locked office where only the researcher has the key. The numbered data will be stored in another locked filing cabinet, i.e., separate from the list with names and numbers. All tests carried out, the registration forms, and the CDIs will be marked with the students subject ID from the randomization list.

The data that will appear on each test will list the number of correct and incorrect responses. The data that will appear on student forms from each work session will list also the correct and incorrect responses as well as the time used. It is particularly important to measure the students work progress, because they have to achieve at least 80% correct responses before they can move on to the next folder.

The data that will be collected on social validity will inform the researchers whether the students have liked working on the reading program and if they would like to continue. For children who use AAC to communicate, assessing consumer satisfaction should involve the AAC user to the greatest extent possible [52]. Directly asking the student about their experiences during and after the intervention may increase intervention relevance to the student and possibly increasing motivation to adhere to and complete the intervention [53]. In connection with data from the teachers’ survey, they will say something about the teachers’ experiences with the reading program and the reading material.

#### Randomization

The four baselines will be randomized using PASS23 via Wei’s urn algorithm that minimizes unbalanced groups. Wei’s urn randomization algorithm dynamically changes the group assignment probabilities based on the degree of imbalance to achieve longitudinal balance between groups [54].

#### Allocation concealment

To protect the assignment sequence until the allocation of when the baseline will start, a “third-party” assignment will be used. A biostatistician will be called every time a student has all the inclusion and exclusion criteria filled, and he will also be responsible for generating the random generation. The enrollment of the student will be done by the first author of this manuscript (LBU).

#### Blinding

This is an open-label trial because it is impossible to mask the students when the intervention starts (baseline). Moreover, given that the first author of this manuscript is the research leader of the project, she will coordinate the field work and will act as one of the assessors based on her training on the assessment of the outcomes and intervention and thus will not be able to remain blinded from the study subjects. Since all the 18 assessments across time will be filmed after the agreement and reliability were checked by two assessors, the teachers will not be blinded on which phase of the intervention the students are assigned. However, the two assessors will code the outcomes independently to establish interobserver agreement on the coded observations.

#### Statistical analysis plans

Generalized estimating equation (GEE) with work matrix of first-order autoregressive factor will be used to the effects of the implementation of the intervention after the baseline assessment. Missing data will be treated as missing at a random mechanism via restricted maximum likelihood. The adopted significance level is 0.0125 (due to the four correlated outcomes, reducing the false discovery rates). For interobserver quality check, we will calculate the interobserver agreement using intraclass correlation (ICC) [55]. There are no plans to conduct additional analyses (e.g., subgroups or adjusted analyses).

#### Video coding

As mentioned earlier, within each phase, 20% of the recorded films will be randomly selected and evaluated by two external raters at item-level across the five primary outcomes. Agreement between the two raters and the teachers will be checked, and in case of agreements below 80%, all the films will be checked. The research assistant will be blinded to when the intervention will start for each student. We have operationalized all aspects of the testing, drew up observation criteria, and made a check list that will be followed when observing the videos. The teachers will record answers during the testing, and the researcher and the researcher assistant score the films blinded for each other on the basis of the checklist in order to calculate the interobserver agreement.

## Discussion

Considering ethical challenges, national and international guidelines such as the National Committee for Research Ethics in the Social Sciences and the Humanities (NESHs) guidelines [56], the Declaration of Helsinki, and the Norwegian Agency for Shared Services in Education and Research (Sikt) have been used as guidelines. The consideration of vulnerable groups is emphasized strongly in the Declaration of Helsinki [57]. While it is open to research on vulnerable groups, and research is seen as a necessity, it is important to be aware that this group can be exposed to risk through, for example, a lack of knowledge and unsafe treatment. There are several ethical challenges that have been discussed. Backe-Hansen [58] points out the relationship between children’s competence and vulnerability as central to ethical assessments. As the participants in this study will be under 14 years of age, have intellectual disabilities, and have no functional speech, it is essential to emphasize that all guidelines to protect the student’s anonymity and integrity will be followed. It can be demanding to ensure anonymity, but in this study, we consider the requirement for anonymization to be extra important. To ensure this, there will be 40 participants from 12 different schools spread over five large municipalities. This is particularly important as several participants may have combinations of characteristics that a narrow selection in a restricted geographical area could have more easily detected.

Studies that include people who cannot give informed, voluntary consent require particularly thorough professional and ethical assessments [59]. Many individuals with intellectual disabilities may have difficulty giving qualified consent, and consent forms will not be distributed to the students. The information must be understood, and informed consent presupposes that one also understands the meaning and what is being informed. However, consenting to something and being willing to do something can be equated. At the same time, it depends on the individual’s ability to know the difference between yes and no [59]. Therefore, the parents will consent on behalf of their children. An information letter and consent form for parents and teachers are designed in line with the Sikt template [60].

Another issue that has been discussed extensively is the presentation and information of and about the students. Prominent research in the field points out weaknesses in many studies as they lack characteristics of students who participate in such studies. This contributes to the fact that it can be challenging to conclude for whom the interventions work. Thus, we will extract and analyze information about factors such as age, gender, diagnosis, IQ/mental age, and communicative skills. Stigmatization, especially disseminating research results, has also been discussed. How students with intellectual disabilities are portrayed in research data is extremely important, so the data and interpretations of data do not intend to stigmatize this group of students.

Qualitative data from the questionnaire to the teachers will help identify barriers to the successful delivery of the intervention. Feedback about how the reading material fits into the school and teaching will be used to enhance the social validity of the intervention.

For students who do not have verbal speech and require AAC, it is necessary to modify and adapt the assessments. In this study, the modifications include having the students point to symbols, letters, and printed words. Modifications like this can fundamentally change the nature of the task [11]. Therefore, all the modifications that will be executed will be done as carefully as possible to support reader’s interpretation of results and not divert away from the initial purpose of the study design and intervention. There is a need for standardized assessments of both phonological awareness and reading that do not acquire speech responses [11].

There is a need for a standard within reading instructions for students with intellectual disabilities who require AAC. Existing research describes a lack of competence, a lack of expectations, and a lack of evidence-based reading programs, which could be adapted for those who require AAC. Adapting instructions, to meet the needs of students who require AAC, are important as it contributes to overall effectiveness of intervention and results in positive literacy gains [16]. The ability to have verbal speech should not factor as the rationale and the foundational for reading interventions [61].

## Trial status

The Protocol ID: 988,724.

Date for submission is March 12. The recruitment began 1 September 2022 and was completed 14 February 2023. The primary study completion is anticipated October 15, 2024. Study completion is anticipated December 18, 2024. We could not submit earlier as several participants were very unsure about participation, and it took time to get signatures on the consent forms.

## Supplementary Information

**Additional file 1.**

## Data Availability

Data sharing is not applicable to this protocol as no datasets were generated or analyzed for this protocol. Participant data will be available at a later date, earliest winter 2025. The datasets analyzed during the current study and statistical code are available from the corresponding author on reasonable request, as is the full protocol. This trial does not involve collecting biological specimens for storage.

## Abbreviations

AAC: Augmentative and alternative communication
ALL: Accessible Literacy Learning
ASD: Autism spectrum disorder
CDI: MacArthur-Bates Communicative Development Inventories
CP: Cerebral palsy
CVC: Consonant-vowel-consonant
DS: Down syndrome
GEE: Generalized estimating equation
ICC: Intraclass correlation
ID: Intellectual disability
NESH: National Committee for Research Ethics in the social Sciences and the Humanities
NRP: National Reading Panel
PASS: Power Analysis and Sample Size software
QED: Quasi-experimental design
RCT: Randomized controlled trial
SIKT: Norwegian Agency for Shared Services in Education and Research
USN: University of South-Eastern of Norway
WWC: What Works Clearinghouse
